# Decomposing Acute Symptom Severity in Large Vessel Occlusion Stroke: Association With Multiparametric CT Imaging and Clinical Parameters

**DOI:** 10.3389/fneur.2021.651387

**Published:** 2021-03-11

**Authors:** Lena Stueckelschweiger, Steffen Tiedt, Daniel Puhr-Westerheide, Matthias P. Fabritius, Franziska Mueller, Lars Kellert, Stefan Maurus, Sergio Grosu, Johannes Rueckel, Moriz Herzberg, Thomas Liebig, Jens Ricke, Konstantinos Dimitriadis, Wolfgang G. Kunz, Paul Reidler

**Affiliations:** ^1^Department of Radiology, University Hospital, Ludwig-Maximilians-University of Munich, Munich, Germany; ^2^Institute for Stroke and Dementia Research, University Hospital, Ludwig-Maximilians-University of Munich, Munich, Germany; ^3^Department of Neurology, University Hospital, Ludwig-Maximilians-University of Munich, Munich, Germany; ^4^Institute of Neuroradiology, University Hospital, Ludwig-Maximilians-University of Munich, Munich, Germany; ^5^Department of Diagnostic and Interventional Radiology, University Hospital Würzburg, Würzburg, Germany

**Keywords:** large vessel occlusion, multiparametric CT, CT perfusion, cerebral ischemia, stroke

## Abstract

**Background and Purpose:** Acute ischemic stroke of the anterior circulation due to large vessel occlusion (LVO) is a multifactorial process, which causes neurologic symptoms of different degree. Our aim was to examine the impact of neuromorphologic and vascular correlates as well as clinical factors on acute symptom severity in LVO stroke.

**Methods:** We selected LVO stroke patients with known onset time from a consecutive cohort which underwent multiparametric CT including non-contrast CT, CT angiography and CT perfusion (CTP) before thrombectomy. Software-based quantification was used to calculate CTP total ischemic and ischemic core volume. Symptom severity was assessed using the National Institutes of Health Stroke Scale (NIHSS) upon admission. Multivariable regression analysis was performed to determine independent associations of admission NIHSS with imaging and clinical parameters. Receiver operating characteristics (ROC) analyses were used to examine performance of imaging parameters to classify symptom severity.

**Results:** We included 142 patients. Linear and ordinal regression analyses for NIHSS and NIHSS severity groups identified significant associations for total ischemic volume [β = 0.31, *p* = 0.01; Odds ratio (OR) = 1.11, 95%-confidence-interval (CI): 1.02–1.19], clot burden score (β = −0.28, *p* = 0.01; OR = 0.76, 95%-CI: 0.64–0.90) and age (β = 0.17, *p* = 0.04). No association was found for ischemic core volume, stroke side, collaterals and time from onset. Stroke topography according to the Alberta Stroke Program CT Score template did not display significant influence after correction for multiple comparisons. AUC for classification of the NIHSS threshold ≥6 by total ischemic volume was 0.81 (*p* < 0.001).

**Conclusions:** We determined total ischemic volume, clot burden and age as relevant drivers for baseline NIHSS in acute LVO stroke. This suggests that not only mere volume but also degree of occlusion influences symptom severity. Use of imaging parameters as surrogate for baseline NIHSS reached limited performance underlining the need for combined clinical and imaging assessment in acute stroke management.

## Introduction

Multiparametric CT imaging raises the opportunity to comprehensively assess cerebrovascular status in large vessel occlusion (LVO) stroke, including tissue perfusion, topography, collateral flow, thrombus burden or edema formation. These parameters directly translate to morphologic correlates of stroke e.g., penumbra and core volume as well as the temporal course of infarction growth (1–5). For functional assessment of stroke severity the in-person examination using the National Institutes of Health Stroke Scale (NIHSS) comprises the gold standard (6). Taken together imaging and NIHSS are the most crucial factors to guide therapy decision for intravenous thrombolysis (IVT) or endovascular therapy (EVT) (7).

While imaging based parameters and baseline NIHSS were extensively studied regarding their impact on chronic outcome after stroke, the interplay of neuromorphologic and vascular stroke correlates with acute symptom severity remains largely unexplored (8–11). Though a study confirmed the intuitive notion that larger ischemia, causes more severe symptoms, ischemic core and penumbra were not differentiated, leaving their effect on acute neurologic symptoms unclear (10). Other studies on later performed or follow-up MRI found an association between stroke topography and admission symptoms, ignoring LVO status or vascular territory, which complicates translation of these results into the modern thrombectomy era (12, 13).

As clinical application, imaging-based surrogates for acute stroke severity might facilitate and accelerate stroke triage. Due to missing neurologic capacities in hospitals, telestroke networks need to implement video assessment for clinical examination (14, 15). Also, during the COVID-19 pandemic, the in-person examination presents a unique challenge, hence it causes prolonged contact with potentially infected and contagious patients (16). On the other hand, MRI or CT perfusion (CTP) imaging, though crucial for decision making in situations with extended or unclear time window, have been shown to increase pretherapeutic time delays (17).

In this study, we aimed to determine how imaging and clinical factors contribute to acute symptom severity of anterior circulation LVO stroke patients as measured on the NIHSS. Further, we explored the possibility to classify guideline-based NIHSS thresholds by imaging parameters.

## Materials and Methods

### Study Design and Cohort

This retrospective study was approved by the local institutional review board according to the Declaration of Helsinki of 2013 and requirement for written informed consent was waived. Patients with acute ischemic stroke due to anterior circulation large vessel occlusion were selected out of a consecutive cohort of 653 patients between 2015 and 2020, who were prospectively enrolled in the German Stroke Registry (clinicaltrials.gov identifier: NCT03356392) and treated with EVT at our institution.

Inclusion criteria were

- Stroke due to anterior circulation large vessel occlusion [internal carotid artery (ICA), middle cerebral artery (MCA)],
- full imaging dataset including non-contrast CT, CT angiography (CTA) and CTP,
- known time from symptom onset.

We excluded patients with

- premorbid modified Rankin Scale ≥2,
- bilateral stroke.

A flowchart of patient selection is provided in the Online Data Supplementary Figure 1.

### Image Analysis/Measurements

**Figure 1:**
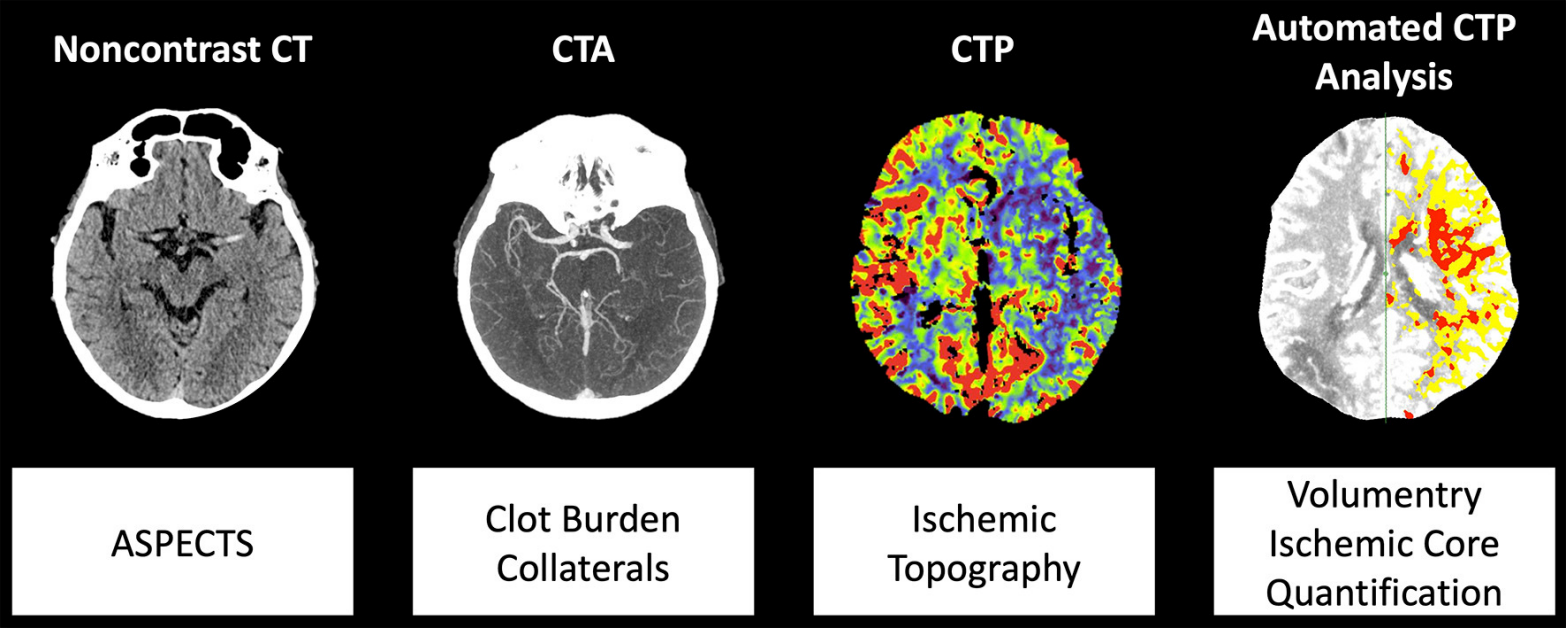
Illustration of multiparametric CT modalities and analyzed parameters. Abbreviations: ASPECTS, Alberta Stroke Program Early CT Score; CTA, CT angiography; CTP, CT perfusion.

The imaging protocol included non-contrast CT, CTA, and CTP. Examinations were performed on CT scanners of the same vendor (SOMATOM Definition AS+, SOMATOM Definition Flash and SOMATOM Force, Siemens Healthineers, Forchheim, Germany). CTP data were processed using Syngo Neuro Perfusion CT (Siemens Healthineers, Forchheim, Germany) including automated calculation of ischemic core and penumbra volumes according to the manufacturer's thresholds (CBV <1.2/100 mL and CBF <35.1/100 mL/min) (18). We defined total ischemic volume as sum of penumbra and core volumes. CTA imaging was obtained in a single sweep from the aortic arch to the vertex with a bolus trigger of 100 HU in the aortic trunk. Expert readers (W.G.K., P.R) blinded to all clinical data including admission NIHSS determined Alberta Stroke Program Early CT Score (ASPECTS) on non-contrast CT as well as regional leptomeningeal (rLM) collateral score according to Menon et al. (20 point ordinal score, higher values indicate better collaterals) and clot burden score according to Tan et al. (10 point ordinal score, smaller values indicate more severe vessel occlusion) on CTA (1, 2, 19). In case of disagreement consensus was reached in a separate session. As measure of inter-reader agreement, we calculated the intraclass correlation coefficient. Stroke topography was assessed on cerebral blood flow maps according to the 10 regions of the ASPECTS template by consensus without double reading. An illustration of multiparametric CT modalities and associated parameters is provided in Figure 1.

### NIHSS and Clinical Parameters

NIHSS was determined for all 142 patients on admission via in-person examination by on call neurologists. Baseline comorbidities were systematically determined after initial therapy by patient interview or medical records with full datasets available in 138 patients.

### Statistical Analysis

Analyses were performed in R version 3.6.2 (R Foundation for Statistical Computing, Vienna Austria) and SPSS Statistics 23 (IBM, Armonk NY 2016, commercial software). Categorical variables are reported as number and percentage. All metric and ordinal variables as median (interquartile range, IQR).

Multivariable linear regression analysis was performed to identify associations of NIHSS with clinical and imaging parameters. To avoid overfitting of the regression models, we tested the multicollinearity of independent variables using the variance inflation factor. For ordinal regression we used a 4-point ordinal scale to classify stroke severity according to the NIHSS (1: minor, NIHSS 0–4; 2: moderate, NIHSS 5–15; 3: moderate to severe, NIHSS: 16–20; 4: severe, NIHSS: 21–42) so that the proportional odds assumption was met (20). Independent model parameters included age, sex, time from symptom onset, stroke side, ischemic core volume, total ischemic volume, non-contrast CT ASPECTS, rLM collateral score and clot burden score. For ordinal regression we used an increment of 10 mL for total ischemic volume in order to facilitate interpretation of the resulting OR.

Additional linear and ordinal regression were performed to incorporate the clinical baseline parameters presence of hypertension, dyslipidemia, diabetes mellitus and atrial fibrillation. Incomplete clinical records led to inclusion of 138 patients in this analysis.

For topography, independent association of hypoperfused ASPECTS regions were tested in separate regression models for each region while adjusting for total ischemic volume and stroke side. *P*-values were corrected using Bonferroni's method.

Receiver operating characteristic (ROC) analyses including calculation of the area under the curve (AUC) were used to determine classification performance of imaging parameters for guideline-based NIHSS thresholds (NIHSS ≥6, ≥10, ≥20, and >25) (21–23). Optimal cut-off values were determined by maximizing the Youden Index. Statistical significance was defined as *p* < 0.05.

## Results

### Patient Characteristics

In this retrospective study 142 patients with LVO stroke were included. Eighty-one of the patients were male and 61 female. Median age was 74 years, [interquartile range (IQR) 63–81 years]. Median time from symptom onset to CT was 124 min (IQR: 70–213 min). Median NIHSS score at admission was 13 (IQR:7–18). Most frequent site of LVO was the M1 segment of the middle cerebral artery (47.9%), followed by the internal carotid artery (25.4%). Median total ischemic volume was 187.5 mL (IQR:152.5–230.7 mL) and median ischemic core volume was 34.8 mL (IQR:21.7–59.1 mL). All patients were treated with EVT according to our inclusion criteria, additionally 102 patients (71.8%) were treated with intravenous thrombolysis. Detailed patient characteristics are displayed in Table 1. Frequency of admission NIHSS values are displayed in the Online Data Supplementary Tables 1, 2.

**Table 1:** Patient characteristics (*N* = 142).

Male sex	81	(57.0%)
Female sex	61	(43.0%)
Median age	74	(63–81)
Male study population	74	(63–80)
Female study population	78	(70–82)
Time from symptom onset to CT (min)	124	(70–213)
NIHSS on admission	13	(7–18)
Hypertension	97	(70.3%)*
Diabetes mellitus	18	(13.0%)*
Hypercholesterolemia	28	(20.3%)*
Atrial Fibrillation	36	(26.1%)*
**Treatment**		
IV thrombolysis	102	(71.8%)
Endovascular therapy	142	(100%)
**Imaging data**		
Noncontrast CT-ASPECTS	9	(7–10)
rLM collateral score	16	(12–18)
Clot burden score	7	(4–8)
Total ischemic volume (mL)	187.5	(152.5–230.7)
Ischemic core volume (mL)	34.8	(21.7–59.1)
Mismatch volume (mL)	61.3	(6.1–82.3)
Infarction growth rate (ml/min)	1.5	(0.8–2.9)
**Side of stroke**		
Right	58	(40.8%)
Left	84	(59.2%)
**Occlusion location**		
ICA	36	(25.4%)
Carotid T	12	(8.5%)
M1 segment of MCA	68	(47.9%)
M2 segment of MCA	26	(18.3%)

Values presented are count (percentage) for categorical and median (interquartile range) for ordinal or continuous variables. Time is presented in minutes, all volumes are presented in mL. ASPECTS, Alberta Stroke Program Early CT Score; ICA, internal carotid artery; MCA, middle cerebral artery; NIHSS, National Institutes of Health Stroke Scale; rLM, regional leptomeningeal. *Available in 138 cases.

### Association of NIHSS With Imaging and Clinical Parameters

Multivariable linear regression analysis in 142 patients presented significant independent association of admission NIHSS with total ischemic volume (β = 0.31, *p* = 0.01), clot burden score (β = −0.28, *p* = 0.01), and age (β = 0.17, *p* = 0.04). Ischemic core volume and rLM collateral score as well as time from symptom onset, non-contrast CT ASPECTS or sex did not show significant associations (all *p* > 0.05). No additional associations were found when including the baseline comorbidities hypertension, diabetes, dyslipidemia or atrial fibrillation (all *p* > 0.05). The variance inflation factor was below the critical value of 3.3 (24).

In ordinal regression analysis with NIHSS values ordered by symptom severity from 1 to 4, significant independent association with total ischemic volume per 10 mL [Odds ratio (OR) = 1.11, 95%-CI: 1.02–1.19, *p* = 0.01] and clot burden score (OR=0.76, 95%-CI: 0.64–0.90, *p* = 0.001) was present. Age and stroke side presented a trend toward significance in this analysis (OR=1.03, 95%-CI: 1.00–1.05, *p* = 0.06 and OR = 2.01, 95%-CI: 0.99–4.06, *p* = 0.05). Detailed results are displayed in Tables 2, 3. Analysis including baseline comorbidities are presented in the Online Data Supplementary Tables 3, 4. Results using the 4-point scale collateral assessment according to Tan et al. and including occlusion location as independent variable are provided in the Online Data Supplementary Tables 5–8 (25). Unadjusted bivariate correlation analysis of admission NIHSS and scaled or ordinal parameters is presented in the Online Data Supplementary Table 9. ICC presented strong agreement for non-contrast CT ASPECTS, rLM collateral score and clot burden score as displayed in the Online Data Supplementary Table 10.

**Table 2:** Linear regression analysis for association of admission NIHSS and imaging parameters (*N* = 142).

**Independent variables**	**β**	***p*-value**	**VIF**
Age	0.17	**0.04**	1.14
Sex	0.04	0.63	1.22
Time from symptom onset to CT	0.06	0.43	1.07
Stroke side	−0.12	0.14	1.13
Core volume	−0.10	0.42	2.56
Total ischemic volume	0.31	**0.01**	2.68
NCCT ASPECTS	−0.11	0.24	1.51
rLM collateral score	0.004	0.97	2.43
Clot burden score	−0.28	**0.01**	1.77

*A multivariate linear regression analysis was performed for the indicated parameters. P-values < 0.05 indicate statistical significance. Bold numbers indicate p < 0.05. VIF, variance inflation factor; ASPECTS, Alberta Stroke Program Early CT Score; NCCT, Noncontrast CT; NIHSS, National Institutes of Health Stroke Scale; rLM, regional leptomeningeal*.

**Table 3:** Ordinal regression analysis for association of symptom severity and imaging parameters (*N* = 142).

**Independent variables**	**OR**	***p*-value**	**95%-CI**
Age	1.03	0.06	1.00-1.05
Sex	0.91	0.79	0.45-1.84
Time from symptom onset to CT	1.00	0.26	1.00-1.00
Stroke side	2.01	0.05	0.99-4.06
Core volume	0.99	0.30	0.98-1.01
Total ischemic volume/10 mL	1.11	**0.01**	1.02-1.19
NCCT ASPECTS	0.90	0.28	0.74-1.09
rLM collateral score	1.01	0.87	0.91-1.13
Clot burden score	0.76	**0.001**	0.64-0.90

*A multivariate ordinal regression analysis was performed for the indicated parameters. Symptom severity was numerically classified by the NIHSS on admission (1: NIHSS 0-4, 2: NIHSS 5-15, 3: NIHSS: 15-20, 4: NIHSS: 21-42). P-values < 0.05 indicate statistical significance. Bold numbers indicate p < 0.05. ASPECTS, Alberta Stroke Program Early CT Score; CI, confidence interval; NCCT, Noncontrast CT; NIHSS, National Institutes of Health Stroke Scale; OR, odds ratio; rLM, regional leptomeningeal*.

### Association of Admission NIHSS With Stroke Topography

Separate regression models for the 10 ASPECTS regions adjusted for total ischemic volume and stroke side resulted in association of lentiform nucleus (β = 0.20, *p* = 0.02) which did not maintain significance after Bonferroni correction for multiple comparisons. Detailed results are presented in the Online Data Supplementary Table 11.

### Discriminatory Value of Imaging Parameters for Guideline-Based NIHSS Thresholds

**Figure 2:**
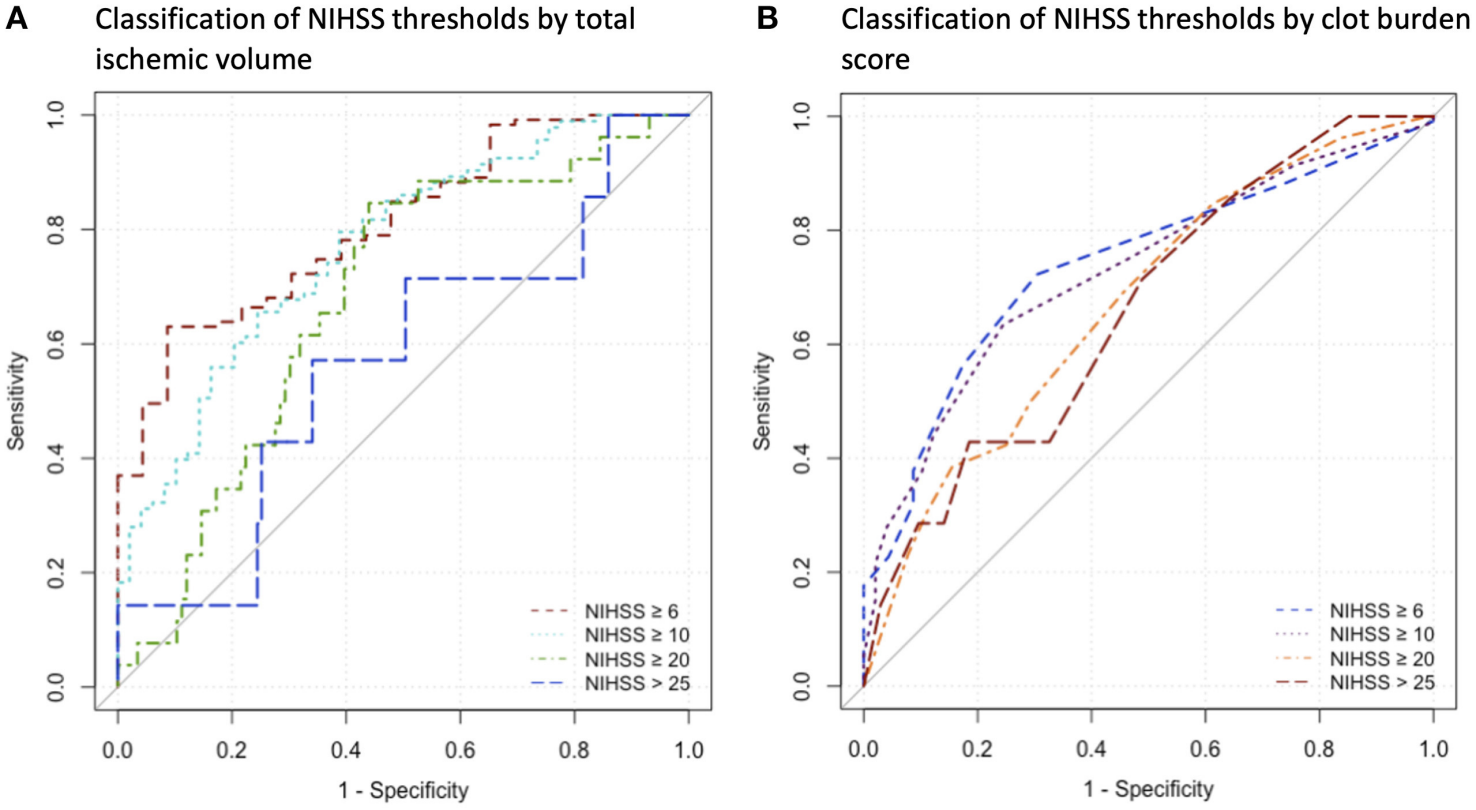
Receiver operating characteristics curves for the indicated parameters. **(A)** Analysis for Classification of NIHSS thresholds by Total Ischemic Volume, **(B)** Analysis for classification of NIHSS thresholds by clot burden score. NIHSS, National Institutes of Health Stroke Scale.

The ROC curve analysis in 142 patients for the discriminatory value of total ischemic volume and clot burden score for the NIHSS threshold ≥6 resulted in an AUC of 0.810 (*p* < 0.001) and 0.74 (*p* < 0.001) respectively, with lower performance for the other thresholds of NIHSS ≥10, ≥20, and >25 (AUC < 0.8). Detailed results are displayed in Table 4. ROC curves are presented in Figure 2.

**Table 4:** ROC analysis for classification of NIHSS thresholds by the indicated parameters (*N* = 142).

**Threshold parameter**	**AUC (95% CI)**		***p*-value**	**Y-Index**	**Y-Index CP**	**Sensitivity**	**Specificity**
**Classification by total ischemic volume (mL)**							
NIHSS ≥ 6	0.81	(0.72–0.89)	<0.001	0.54	182.6 mL	0.63	0.91
NIHSS ≥ 10	0.77	(0.69–0.85)	<0.001	0.42	185.9 mL	0.66	0.76
NIHSS ≥ 20	0.68	(0.57–0.78)	0.001	0.41	185.9 mL	0.85	0.56
NIHSS > 25	0.57	(0.33–0.81)	0.569	0.23	208.7 mL	0.57	0.66
**Classification by clot burden score**							
NIHSS ≥ 6	0.74	(0.64–0.84)	<0.001	0.42	8	0.72	0.70
NIHSS ≥ 10	0.73	(0.65–0.81)	<0.001	0.39	7	0.63	0.76
NIHSS ≥ 20	0.67	(0.56–0.78)	0.003	0.24	7	0.69	0.54
NIHSS > 25	0.66	(0.46–0.87)	0.125	0.24	4	0.43	0.81

*A receiver operating characteristic analysis was performed for the indicated parameters. Cut points were determined by the Y-Index. P-values < 0.05 indicate statistical significance. AUC, Area Under the Curve; CI, confidence interval; CP, Cut point; NIHSS, National Institutes of Health Stroke Scale; Y-Index, Youden Index*.

## Discussion

This study presents a comprehensive investigation of multiparametric CT imaging and clinical factors on acute symptom severity in anterior circulation LVO stroke. To our knowledge this is the first study selectively analyzing impact of CTP ischemic core and total ischemic volume while also providing data on the influence of ischemic topography using the routinely applicable ASPECTS template.

Our results indicate that total ischemic volume is a major determinant for acute symptom severity in LVO patients measured by the NIHSS. Notably, there is also an established linear relationship between NIHSS after 24 h and post-treatment infarction volume hinting toward similar mechanisms moderating morphology and symptom severity in the hyperacute and post-therapeutic acute stroke phase (26). We also observed significant association of the clot burden score with NIHSS on admission similar to other studies (8, 9). Occlusion location did not present independent influence. As possible mechanism we propose that not only ischemic volume but also degree of hypoperfusion, reflected by more severe occlusion, affects acute symptom severity. An interaction that has already been described for initial perfusion deficit and chronic outcome after stroke (27).

Interestingly, neither non-contrast CT ASPECTS nor ischemic core volume exhibited an independent association with symptom severity. As these parameters are closely intertwined, both are particularly useful for predicting morphological and clinical outcomes, yet the effect on acute symptoms seems to be clouded by mere total ischemic volume (28). It is important to note that the inclusion criteria of known time from symptom onset shifted our sample toward an earlier time window. Our study did not reveal correlation of NIHSS and time from symptom onset, though there are observations that NIHSS can worsen during the subacute course of stroke (29). Therefore, our data indicates that the established associations are independent of a defined time window. Later time windows or patients with unknown onset time, however, might still display different dynamics in the translation of ischemic volumes to neurologic symptoms.

Also, collateral status did not impact baseline NIHSS, which is known to affect infarction core growth (5). Accordingly, collateral perfusion would seem to suffice for delaying tissue decline but not for sustaining neurologic function in the acute stroke phase. However, diverging results were found by other studies using less granular collateral scores and disregarding ischemic core volume (9, 30).

Though the NIHSS is known to favor symptoms of the dominant hemisphere, infarction side did not show significant association with symptom severity. These results are in congruence with the findings of Furlanis et al. who found a strong correlation of ischemic volume with baseline NIHSS resulting in similar slopes for right and left hemispheric stroke (10). However, advanced MRI analysis showed left hemispheric stroke led to higher baseline NIHSS (31). Differences in results might partly be explained by the selected study sample containing only LVO patients, requesting dedicated analysis in this important patient group. Also, stroke topography did not significantly impact overall admission NIHSS. Topography seems crucial for chronic stroke symptoms and though there is described location dependency of baseline NIHSS, same locations showed high overlap with lesion volume, which was accounted for in our analysis (13, 15).

Among clinical parameters only age displayed a significant influence on baseline symptom severity with lower NIHSS in younger patients as also found in a prior study (9). Neither sex, nor baseline comorbidities exhibited significant influence.

As potential clinical use, total ischemic volume classified the guideline-based cut-off of admission NIHSS ≥ 6 as used in the DEFUSE 3 study with acceptable performance. Other NIHSS cut-off values of 25 from the WAKE-UP trial or 10 and 20 from the DAWN trial were classified with lower performance (22, 23, 32). Accordingly, only differentiation of minor/moderate stroke by total ischemic volume seams feasible. This underlines the importance of proper clinical and imaging examination for evidence-based therapy decisions.

Limitation of this study include the small sample size and retrospective study design. Second, we used a selected dataset of LVO stroke patients with known time from symptom onset and without premorbid disability (pMRS ≤ 1). We chose these criteria as we wanted to examine the temporal effects on stroke symptoms during LVO without bias of existing sequelae. Translatability to patients with unknown onset seems reasonable due to the missing impact of time from symptom onset, however, needs further dedicated validation. Also, subgroups with advanced edema formation indicated by low ASPECTS and presumably larger infarction core were underrepresented in our study, requiring further analysis of this highly discussed subgroup in larger samples (33). In light of the small sample size also the borderline non-significant ordinal regression results for impact of ischemic core volume and stroke side need to be interpreted with caution and require reproduction in larger datasets. Third, imaging was performed using scanners and software of a single vendor (Siemens Healthineers, Forchheim, Germany). While volumetry can differ between different software packages, measurements of the used package presented best agreement with the gold-standard RAPID among other packages (18, 34).

## Conclusion

Our data determined total ischemic volume and clot burden as the most relevant neuromorphologic and vascular correlates for baseline NIHSS in acute LVO stroke, suggesting that not only mere volume but also degree of occlusion influences clinical presentation. On the other hand, ischemic core volume and collateral status did not influence acute symptom severity. All associations were independent from time from symptom onset. Our results indicate only limited potential for classification of symptom severity by CT imaging. This highlights the significance and synergy of clinical and imaging assessment in the acute management of stroke patients. Further studies with a larger dataset are needed to clarify the role of collaterals and side of stroke.

## Data Availability Statement

The data supporting the conclusions of this article will be made available by the corresponding author upon request.

## Ethics Statement

The studies involving human participants were reviewed and approved by Ethikkomission der medizinischen Fakultät, LMU Munich. Written informed consent for participation was not required for this study in accordance with the national legislation and the institutional requirements.

## Author Contributions

LS, ST, WK, and PR: conceptualization. LS, ST, FM, WK, and PR: formal analysis and data curation. LS, ST, WK, PR, and MF: writing—original draft. PR, DP-W, SG, SM, LK, MH, JR, and KD: writing—review and editing. PR, WK, JR, and TL: supervision. All authors: contributed to the article and approved the submitted version.

## Conflict of Interest

The authors declare that the research was conducted in the absence of any commercial or financial relationships that could be construed as a potential conflict of interest.

## References

[1] Tan IY Demchuk AM Hopyan J Zhang L Gladstone D Wong K. CT angiography clot burden score and collateral score: correlation with clinical and radiologic outcomes in acute middle cerebral artery infarct. AJNR Am J Neuroradiol. (2009) 30:525–31. 10.3174/ajnr.A1408 19147716 PMC7051470

[2] Menon BK Smith EE Modi J Patel SK Bhatia R Watson TW. Regional leptomeningeal score on CT angiography predicts clinical and imaging outcomes in patients with acute anterior circulation occlusions. AJNR Am J Neuroradiol. (2011) 32:1640–5. 10.3174/ajnr.A2564 21799045 PMC7965388

[3] Broocks G Flottmann F Ernst M Faizy TD Minnerup J Siemonsen S. Computed tomography-based imaging of voxel-wise lesion water uptake in ischemic brain: relationship between density and direct volumetry. Invest Radiol. (2018) 53:207–13. 10.1097/RLI.0000000000000430 29200013

[4] Mokin M Levy EI Siddiqui AH Goyal M Nogueira RG Yavagal DR. Association of clot burden score with radiographic and clinical outcomes following Solitaire stent retriever thrombectomy: analysis of the SWIFT PRIME trial. J Neurointerv Surg. (2017) 9:929–32. 10.1136/neurintsurg-2016-012631 27634952

[5] Puhr-Westerheide D Tiedt S Rotkopf LT Herzberg M Reidler P Fabritius MP. Clinical and imaging parameters associated with hyperacute infarction growth in large vessel occlusion stroke. Stroke. (2019) 50:2799–804. 10.1161/STROKEAHA.119.025809 31426729

[6] Lyden P Brott T Tilley B Welch KM Mascha EJ Levine S. Improved reliability of the NIH stroke scale using video training. NINDS TPA Stroke Study Group Stroke. (1994) 25:2220–6. 10.1161/01.STR.25.11.2220 7974549

[7] Powers WJ Rabinstein AA Ackerson T Adeoye OM Bambakidis NC Becker K. Guidelines for the early management of patients with acute ischemic stroke: 2019 update to the 2018 guidelines for the early management of acute ischemic stroke: a guideline for healthcare professionals from the American Heart Association/American Stroke Association. Stroke. (2019) 50:e344–418. 10.1161/STR.0000000000000211 31662037

[8] Fischer U Arnold M Nedeltchev K Brekenfeld C Ballinari P Remonda L. NIHSS score and arteriographic findings in acute ischemic stroke. Stroke. (2005) 36:2121–5. 10.1161/01.STR.0000182099.04994.fc 16151026

[9] Fanou EM Knight J Aviv RI Hojjat SP Symons SP Zhang L. Effect of Collaterals on clinical presentation, baseline imaging, complications, and outcome in acute stroke. AJNR Am J Neuroradiol. (2015) 36:2285–91. 10.3174/ajnr.A4453 26471754 PMC7964273

[10] Furlanis G Ajcevic M Stragapede L Lugnan C Ridolfi M Caruso P. Ischemic volume and neurological deficit: correlation of computed tomography perfusion with the national institutes of health stroke scale score in acute ischemic stroke. J Stroke Cerebrovasc Dis. (2018) 27:2200–7. 10.1016/j.jstrokecerebrovasdis.2018.04.003 29724610

[11] Meyer L Broocks G Bechstein M Flottmann F Leischner H Brekenfeld C. Early clinical surrogates for outcome prediction after stroke thrombectomy in daily clinical practice. J Neurol Neurosurg Psychiatry. (2020) 91:1055–9. 10.1136/jnnp-2020-323742 32934109

[12] Menezes NM Ay H Wang Zhu M Lopez CJ Singhal AB Karonen JO. The real estate factor: quantifying the impact of infarct location on stroke severity. Stroke. (2007) 38:194–7. 10.1161/01.STR.0000251792.76080.45 17122428

[13] Payabvash S Taleb S Benson JC Mckinney AM. Acute ischemic stroke infarct topology: association with lesion volume and severity of symptoms at admission and discharge. AJNR Am J Neuroradiol. (2017) 38:58–63. 10.3174/ajnr.A4970 27758775 PMC7963653

[14] Demaerschalk BM Vegunta S Vargas BB Wu Q Channer DD Hentz JG. Reliability of real-time video smartphone for assessing National Institutes of Health Stroke Scale scores in acute stroke patients. Stroke. (2012) 43:3271–7. 10.1161/STROKEAHA.112.669150 23160878

[15] Etherton MR Rost NS Wu O. Infarct topography and functional outcomes. J Cereb Blood Flow Metab. (2018) 38:1517–32. 10.1177/0271678X17700666 28345373 PMC6125960

[16] Qureshi AI Abd-Allah F Al-Senani F Aytac E Borhani-Haghighi A Ciccone A. Management of acute ischemic stroke in patients with COVID-19 infection: report of an international panel. Int J Stroke. (2020) 15:540–54. 10.1177/1747493020923234 32362244

[17] Goyal M Jadhav AP Bonafe A Diener H Mendes Pereira V Levy E. Analysis of workflow and time to treatment and the effects on outcome in endovascular treatment of acute ischemic stroke: results from the SWIFT PRIME randomized controlled trial. Radiology. (2016) 279:888–97. 10.1148/radiol.2016160204 27092472

[18] Austein F Riedel C Kerby T Meyne J Binder A Lindner T. Comparison of perfusion CT software to predict the final infarct volume after thrombectomy. Stroke. (2016) 47:2311–7. 10.1161/STROKEAHA.116.013147 27507864

[19] Pexman JH Barber PA Hill MD Sevick RJ Demchuk AM Hudon ME. Use of the alberta stroke program early CT score (ASPECTS) for assessing CT scans in patients with acute stroke. AJNR Am J Neuroradiol. (2001) 22:1534–42. 11559501 PMC7974585

[20] Hage V. The NIH stroke scale: a window into neurological status. NurseCom Nursing Spectrum (Greater Chicago). (2011) 24:44–9.

[21] Albers GW Marks MP Kemp S Christensen S Tsai JP Ortega-Gutierrez S. Thrombectomy for stroke at 6 to 16 hours with selection by perfusion imaging. N Engl J Med. (2018) 378:708–18. 10.1056/NEJMoa1713973 29364767 PMC6590673

[22] Nogueira RG Jadhav AP Haussen DC Bonafe A Budzik RF Bhuva P. Thrombectomy 6 to 24 hours after stroke with a mismatch between deficit and infarct. N Engl J Med. (2018) 378:11–21. 10.1056/NEJMoa1706442 29129157

[23] Thomalla G Simonsen CZ Boutitie F Andersen G Berthezene Y Cheng B. MRI-guided thrombolysis for stroke with unknown time of onset. N Engl J Med. (2018) 379:611–22. 10.1056/NEJMoa1804355 29766770

[24] Kock N Lynn G. Lateral collinearity and misleading results in variance-based SEM: an illustration and recommendations. Econom Mult Equation Models eJournal. (2012) 13:546–80. 10.17705/1jais.00302

[25] Tan JC Dillon WP Liu S Adler F Smith WS Wintermark M. Systematic comparison of perfusion-CT and CT-angiography in acute stroke patients. Ann Neurol. (2007) 61:533–43. 10.1002/ana.21130 17431875

[26] Al-Ajlan FS Al Sultan AS Minhas P Assis Z De Miquel MA Millan M. Posttreatment infarct volumes when compared with 24-hour and 90-day clinical outcomes: insights from the REVASCAT randomized controlled trial. AJNR Am J Neuroradiol. (2018) 39:107–10. 10.3174/ajnr.A5463 29170266 PMC7410691

[27] Rotkopf LT Tiedt S Puhr-Westerheide D Herzberg M Reidler P Kellert L. Ischemic core volume combined with the relative perfusion ratio for stroke outcome prediction after endovascular thrombectomy. J Neuroimaging. (2020) 30:321–6. 10.1111/jon.12695 32037660

[28] Kim-Tenser M Mlynash M Lansberg MG Tenser M Bulic S Jagadeesan B. CT perfusion core and ASPECT score prediction of outcomes in DEFUSE 3. Int J Stroke. (2020) 1747493020915141. 10.1177/1747493020915141 32233746

[29] Degraba TJ Hallenbeck JM Pettigrew KD Dutka AJ Kelly BJ. Progression in acute stroke: value of the initial NIH stroke scale score on patient stratification in future trials. Stroke. (1999) 30:1208–12. 10.1161/01.STR.30.6.1208 10356101

[30] Marks MP Lansberg MG Mlynash M Olivot JM Straka M Kemp S. Effect of collateral blood flow on patients undergoing endovascular therapy for acute ischemic stroke. Stroke. (2014) 45:1035–9. 10.1161/STROKEAHA.113.004085 24569816 PMC4396867

[31] Wu O Cloonan L Mocking SJ Bouts MJ Copen WA Cougo-Pinto PT. Role of acute lesion topography in initial ischemic stroke severity and long-term functional outcomes. Stroke. (2015) 46:2438–44. 10.1161/STROKEAHA.115.009643 26199314 PMC4550548

[32] Albers GW Lansberg MG Kemp S Tsai JP Lavori P Christensen S. A multicenter randomized controlled trial of endovascular therapy following imaging evaluation for ischemic stroke (DEFUSE 3). Int J Stroke. (2017) 12:896–905. 10.1177/1747493017701147 28946832 PMC5916787

[33] Kaesmacher J Chaloulos-Iakovidis P Panos L Mordasini P Michel P Hajdu SD. Mechanical thrombectomy in ischemic stroke patients with alberta stroke program early computed tomography score 0-5. Stroke. (2019) 50:880–8. 10.1161/STROKEAHA.118.023465 30827193 PMC6430594

[34] Koopman MS Berkhemer OA Geuskens R Emmer BJ Van Walderveen MAA. Comparison of three commonly used CT perfusion software packages in patients with acute ischemic stroke. J Neurointerv Surg. (2019) 11:1249–56. 10.1136/neurintsurg-2019-014822 31203208

