# Supplementary material for: Decomposing Acute Symptom Severity in Large Vessel Occlusion Stroke: Association With Multiparametric CT Imaging and Clinical Parameters
Source: Front Neurol. 2021 Mar 11;12:651387. doi: 10.3389/fneur.2021.651387 (PMC7991695; doi:10.3389/fneur.2021.651387)
Supplement: Supplementary file 1 [file Data_Sheet_1.docx]

Supplementary Material


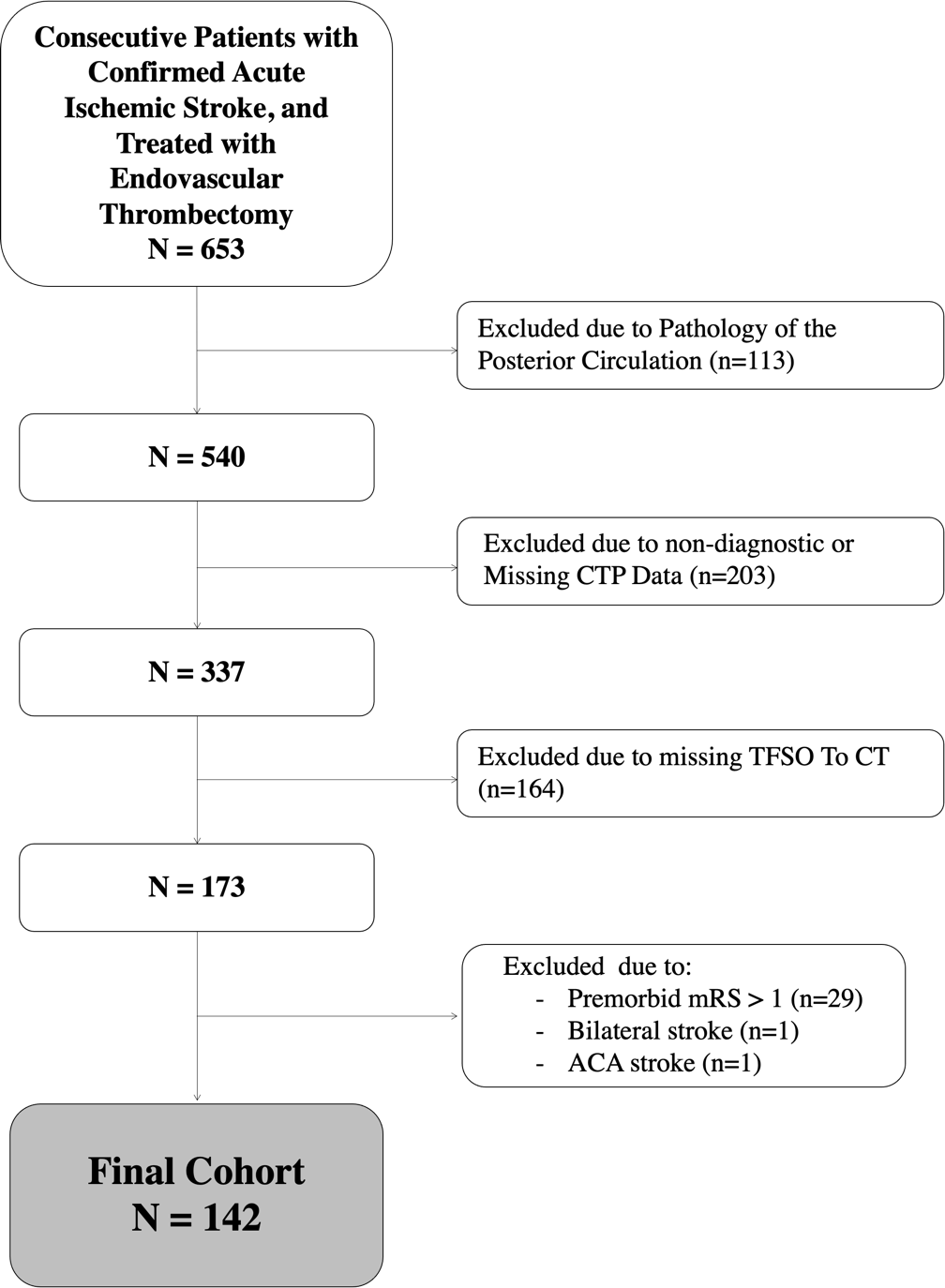


**Supplemental Figure 1. Flow chart of patient selection.** CTP indicates CT perfusion, TFSO indicates Time from Symptom Onset, mRS indicates modified Rankin Scale, ACA indicates arteria cerebri anterior.

| **Supplemental Table 1. NIHSS score frequency (N = 142).** | | |
| --- | --- | --- |
| **NIHSS Score** | **n** | **%** |
| 0 | 2 | 1.41% |
| 1 | 2 | 1.41% |
| 2 | 3 | 2.11% |
| 3 | 6 | 4.23% |
| 4 | 6 | 4.23% |
| 5 | 4 | 2.82% |
| 6 | 7 | 4.93% |
| 7 | 7 | 4.93% |
| 8 | 6 | 4.23% |
| 9 | 6 | 4.23% |
| 10 | 11 | 7.75% |
| 11 | 3 | 2.11% |
| 12 | 6 | 4.23% |
| 13 | 5 | 3.52% |
| 14 | 11 | 7.75% |
| 15 | 3 | 2.11% |
| 16 | 9 | 6.34% |
| 17 | 9 | 6.34% |
| 18 | 9 | 6.34% |
| 19 | 1 | 0.70% |
| 20 | 4 | 2.82% |
| 21 | 4 | 2.82% |
| 22 | 6 | 4.23% |
| 23 | 3 | 2.11% |
| 24 | 1 | 0.70% |
| 25 | 1 | 0.70% |
| 27 | 1 | 0.70% |
| 28 | 1 | 0.70% |
| 29 | 1 | 0.70% |
| 30 | 1 | 0.70% |
| 32 | 1 | 0.70% |
| 33 | 2 | 1.41% |
| *Distribution of NIHSS-Score, Values presented are count and percentage. Abbreviations: NIHSS, National Institutes of Health Stroke Scale.* | | |

| **Supplemental Table 2. NIHSS Subsets frequency (N = 142).** | | | |
| --- | --- | --- | --- |
| **NIHSS score** | **NIHSS Subsets** | **n** | **%** |
| 0 - 4 | No/Minor Stroke | 19 | 13.4% |
| 5 - 15 | Moderate Stroke | 69 | 48.6% |
| 16 - 20 | Moderate to severe Stroke | 32 | 22.5% |
| 21 - 42 | Severe Stroke | 22 | 15.5% |
| *Distribution of NIHSS Subsets, Values presented are count and percentage. Abbreviations: NIHSS, National Institutes of Health Stroke Scale.* | | | |

| **Supplemental Table 3. Linear regression analysis for association of admission NIHSS with imaging and clinical parameters (n=138)*.** | | | |
| --- | --- | --- | --- |
|  | **Admission**  **NIHSS** | | |
| **Independent variables** | **β** | **p-value** | **VIF** |
| Age | 0.22 | **0.01** | 1.28 |
| Sex | 0.04 | 0.67 | 1.26 |
| Time from Symptom Onset to CT | 0.04 | 0.63 | 1.09 |
| Stroke Side | -0.08 | 0.33 | 1.17 |
| Core volume | -0.09 | 0.44 | 2.42 |
| Total ischemic volume | 0.29 | **0.02** | 2.46 |
| NCCT ASPECTS | -0.14 | 0.16 | 1.57 |
| rLM collateral score | 0.03 | 0.82 | 2.42 |
| Clot Burden Score | -0.22 | **0.03** | 1.76 |
| Arterial Hypertension | -0.15 | 0.10 | 1.32 |
| Diabetes mellitus | 0.01 | 0.89 | 1.13 |
| Dyslipidemia | 0.07 | 0.43 | 1.16 |
| Atrial fibrillation | -0.002 | 0.98 | 1.11 |
| *A multivariate linear regression analysis was performed for the indicated parameters. Bold numbers indicate p < 0.05. Abbreviations: VIF, variance inflation factor; ASPECTS, Alberta Stroke Program Early CT Score; NCCT, Noncontrast CT and NIHSS, National Institutes of Health Stroke Scale and rLM, regional leptomeningeal. *Full dataset available in 138/142 patients.* | | | |

| **Supplemental Table 4. Ordinal regression analysis for association of admission NIHSS with imaging and clinical parameters (n=138)*.** | | | | | |
| --- | --- | --- | --- | --- | --- |
| **Independent variables** | **OR** | **p-value** | **95%-CI** | | |
| Age | 1.03 | **0.05** | 1.00 | - | 1.06 |
| Sex | 0.86 | 0.68 | 0.41 | - | 1.79 |
| Time from symptom onset to CT | 1.00 | 0.35 | 1.00 | - | 1.00 |
| Stroke Side | 1.87 | 0.09 | 0.90 | - | 3.87 |
| Core volume | 1.00 | 0.36 | 0.98 | - | 1.01 |
| Total ischemic volume | 1.01 | **0.02** | 1.00 | - | 1.02 |
| NCCT ASPECTS | 0.88 | 0.21 | 0.73 | - | 1.07 |
| rLM collateral score | 1.01 | 0.84 | 0.91 | - | 1.13 |
| Clot Burden Score | 0.80 | **0.01** | 0.67 | - | 0.95 |
| Arterial Hypertension | 1.85 | 0.14 | 0.82 | - | 4.15 |
| Diabetes mellitus | 0.65 | 0.41 | 0.23 | - | 1.84 |
| Dyslipidemia | 0.70 | 0.41 | 0.30 | - | 1.65 |
| Atrial fibrillation | 1.05 | 0.91 | 0.48 | - | 2.28 |
| *A multivariate ordinal regression analysis was performed for the indicated parameters. Symptom severity was numerically classified by the NIHSS on admission (1: NIHSS 0-4, 2: NIHSS 5-15, 3: NIHSS: 15-20, 4: NIHSS: 21-42). Bold numbers indicate p < 0.05. Abbreviations: ASPECTS, Alberta Stroke Program Early CT Score; CI, confidence interval; NCCT, Noncontrast CT; NIHSS, National Institutes of Health Stroke Scale; OR, odds ratio and rLM, regional leptomeningeal. *Full dataset available in 138/142 patients.* | | | | | |

| **Supplemental Table 5. Linear regression analysis for association of admission NIHSS including the Tan et al. collateral scale (N = 142).** | | | |
| --- | --- | --- | --- |
|  | **Admission**  **NIHSS** | | |
| **Independent variables** | **β** | **p-value** | **VIF** |
| Age | 0.17 | **0.03** | 1.08 |
| Sex | 0.04 | 0.62 | 1.09 |
| Time from Symptom Onset to CT | 0.06 | 0.43 | 1.07 |
| Stroke Side | 0.12 | 0.13 | 1.07 |
| Core volume | -0.10 | 0.41 | 2.51 |
| Total ischemic volume | 0.31 | **0.01** | 2.42 |
| NCCT ASPECTS | -0.11 | 0.23 | 1.48 |
| Tan et al. collateral scale | 0.06 | 0.95 | 1.26 |
| Clot Burden Score | -0.28 | **0.01** | 1.65 |
| *A multivariate linear regression analysis was performed for the indicated parameters. Bold numbers indicate p < 0.05. Abbreviations: VIF, variance inflation factor; ASPECTS, Alberta Stroke Program Early CT Score; NCCT, Noncontrast CT and NIHSS, National Institutes of Health Stroke Scale.* | | | |

| **Supplemental Table 6. Ordinal regression analysis for association of admission NIHSS including the Tan et al. collateral scale (N = 142).** | | | | | |
| --- | --- | --- | --- | --- | --- |
| **Independent variables** | **OR** | **p-value** | **95%-CI** | | |
| Age | 1.02 | 0.07 | 1.00 | - | 1.05 |
| Sex | 0.80 | 0.46 | 0.43 | - | 1.46 |
| Time from Symptom Onset to CT | 1.00 | 0.81 | 1.00 | - | 1.00 |
| Stroke Side | 0.61 | 0.11 | 0.33 | - | 1.11 |
| Total ischemic volume | 1.11 | **0.01** | 1.02 | - | 1.20 |
| Core volume | 1.00 | 0.26 | 0.99 | - | 1.00 |
| NCCT ASPECTS | 0.88 | 0.16 | 0.75 | - | 1.05 |
| Tan et al. collateral scale | 0.95 | 0.83 | 0.60 | - | 1.51 |
| Clot Burden Score | 0.79 | **0.01** | 0.68 | - | 0.92 |
| *A multivariate ordinal regression analysis was performed for the indicated parameters. Symptom severity was numerically classified by the NIHSS on admission (1: NIHSS 0-4, 2: NIHSS 5-15, 3: NIHSS: 15-20, 4: NIHSS: 21-42). Bold numbers indicate p < 0.05. Abbreviations: ASPECTS, Alberta Stroke Program Early CT Score; CI, confidence interval; NCCT, Noncontrast CT; NIHSS, National Institutes of Health Stroke Scale; OR, odds ratio and rLM, regional leptomeningeal.* | | | | | |

| **Supplemental Table 7. Linear regression analysis for association of admission NIHSS including occlusion location (N = 142).** | | | |
| --- | --- | --- | --- |
|  | **Admission**  **NIHSS** | | |
| **Independent variables** | **β** | **p-value** | **VIF** |
| Age | 0.17 | **0.04** | 1.20 |
| Sex | 0.04 | 0.63 | 1.22 |
| Time from Symptom Onset to CT | 0.06 | 0.44 | 1.08 |
| Stroke Side | 0.13 | 0.12 | 1.16 |
| Total ischemic volume | 0.29 | **0.02** | 2.91 |
| Core volume | -0.07 | 0.57 | 2.79 |
| NCCT ASPECTS | -0.11 | 0.26 | 1.52 |
| rLM collateral score | 0.02 | 0.88 | 2.50 |
| Clot Burden Score | -0.26 | **0.02** | 2.26 |
| ICA / CART occlusion | -0.01 | 0.91 | 1.58 |
| M1 occlusion | -0.04 | 0.77 | 2.84 |
| M2 occlusion | -0.10 | 0.44 | 2.84 |
| *A multivariate linear regression analysis was performed for the indicated parameters. Bold numbers indicate p < 0.05. Abbreviations: VIF, variance inflation factor; ASPECTS, Alberta Stroke Program Early CT Score; NCCT, Noncontrast CT and NIHSS, National Institutes of Health Stroke Scale and rLM, regional leptomeningeal.* | | | |

| **Supplemental Table 8. Ordinal regression analysis for association of admission NIHSS including occlusion location (N = 142).** | | | | | |
| --- | --- | --- | --- | --- | --- |
| **Independent variables** | **OR** | **p-value** | **95%-CI** | | |
| Age | 1.03 | 0.07 | 1.00 | - | 1.05 |
| Sex | 0.90 | 0.77 | 0.44 | - | 1.83 |
| Time from Symptom Onset to CT | 1.00 | 0.25 | 1.00 | - | 1.01 |
| Stroke Side | 0.49 | 0.05 | 0.24 | - | 1.00 |
| Total ischemic volume | 1.11 | **0.01** | 1.02 | - | 1.20 |
| Core volume | 1.00 | 0.34 | 0.98 | - | 1.01 |
| NCCT ASPECTS | 0.90 | 0.29 | 0.75 | - | 1.09 |
| rLM collateral score | 1.01 | 0.81 | 0.91 | - | 1.13 |
| Clot Burden Score | 0.78 | **0.01** | 0.64 | - | 0.95 |
| ICA / CART occlusion | 1.39 | 0.62 | 0.39 | - | 5.01 |
| M1 occlusion | 1.26 | 0.73 | 0.35 | - | 4.55 |
| M2 occlusion | 1.77 | 0.49 | 0.35 | - | 8.94 |
| *A multivariate ordinal regression analysis was performed for the indicated parameters. Symptom severity was numerically classified by the NIHSS on admission (1: NIHSS 0-4, 2: NIHSS 5-15, 3: NIHSS: 15-20, 4: NIHSS: 21-42). Bold numbers indicate p < 0.05. Abbreviations: ASPECTS, Alberta Stroke Program Early CT Score; CI, confidence interval; NCCT, Noncontrast CT; NIHSS, National Institutes of Health Stroke Scale; OR, odds ratio and rLM, regional leptomeningeal.* | | | | | |

| **Supplemental Table 9. Bivariate Correlation of indicated parameters with admission NIHSS (N = 142).** | | |
| --- | --- | --- |
|  | **Pearson correlation coefficient** | **p-value** |
| Age | 0.15 | 0.81 |
| Time from symptom onset to CT | 0.06 | 0.95 |
| Core volume | 0.28 | **0.001** |
| Total ischemic volume | 0.39 | **<0.001** |
| NCCT ASPECTS | 0.31 | **<0.001** |
| rLM collateral score | 0.34 | **<0.001** |
| Clot Burden Score | 0.41 | **<0.001** |
| *Bold numbers indicate p < 0.05. Abbreviations: ASPECTS, Alberta Stroke Program Early CT Score; NCCT, Noncontrast CT; NIHSS, National Institutes of Health Stroke Scale; OR, odds ratio and rLM, regional leptomeningeal.* | | |

| **Supplemental Table 10. Inter-Reader Agreement (N = 142).** | | |
| --- | --- | --- |
|  | **Intraclass correlation coefficient** | **(95%-CI)** |
| NCCT ASPECTS | 0.72 | (0.69-0.90) |
| rLM collateral score | 0.88 | (0.83-0.91) |
| Clot burden score | 0.92 | (0.89-0.94) |
| *Abbreviations: ASPECTS, Alberta Stroke Program Early CT Score; CI, confidence interval; NCCT, Noncontrast CT and rLM, regional leptomeningeal.* | | |

| **Supplemental Table 11. Association of NIHSS with Acute Stroke Topography (N = 142).** | | | |
| --- | --- | --- | --- |
| **Independent variables** | **β** | **p-value** | **VIF** |
| Caudate Nucleus | 0.15 | 0.07 | 1.25 |
| Internal Capsule | 0.14 | 0.10 | 1.25 |
| Insula | 0.08 | 0.33 | 1.05 |
| Lentiform Nucleus | 0.20 | 0.02 | 1.25 |
| M1 Cortex | 0.01 | 0.86 | 1.11 |
| M2 Cortex | - 0.04 | 0.63 | 1.12 |
| M3 Cortex | - 0.05 | 0.53 | 1.17 |
| M4 Cortex | 0.04 | 0.63 | 1.07 |
| M5 Cortex | - 0.56 | 0.57 | 1.02 |
| M6 Cortex | 0.15 | 0.054 | 1.08 |
| A linear logistic regression analysis was performed for the indicated parameters adjusted for total ischemic volume and stroke side. *Abbreviations: VIF, variance inflation factor; NIHSS, National Institutes of Health Stroke Scale and M1-M6, cortical regions of the ASPECTS score.* | | | |
